# Drug-induced coagulopathies: a real-world pharmacovigilance study using the FDA adverse event reporting system

**DOI:** 10.3389/fphar.2024.1486422

**Published:** 2024-12-18

**Authors:** Yanjun Lu, Qian Xu, Shita Zhu

**Affiliations:** Pharmacy Department, Xiamen Fifth Hospital, Xiamen, Fujian, China

**Keywords:** coagulopathies, FAERS database, adverse event, data mining, disproportionality analyses, pharmacovigilance

## Abstract

**Background:**

This study aims to investigate adverse drug reaction signals associated with coagulopathies through data mining using the Adverse Event Reporting System (FAERS) of the US Food and Drug Administration. Prompt identification of high-risk drugs provides a valuable basis for enhancing clinical drug safety.

**Methods:**

The adverse event reports related to coagulopathies from Q1 2004 to Q2 2024 were extracted from the ASCII data packages in FAERS. The reporting odds ratio (ROR), proportional reporting ratio (PRR), and Bayesian confidence propagation neural network (BCPNN) were used to identify adverse drug reaction signals associated with coagulopathies.

**Results:**

During the reporting period, 40,545 reports were retrieved, with a slightly higher proportion of females than males. Among the top 30 drugs associated with the occurrence of coagulopathies, 24 drugs exhibited positive signals in risk analysis. Based on the individual drug reporting odds ratio (95% confidence interval) as a measure of risk signal strength, the top five drugs are as follows: gemcitabine [ROR (95% CI):16.87 (15.83–17.98)], busulfan [ROR (95% CI):15.51 (13.69–17.58)], anti-thymocyte globulin [ROR (95% CI):15.49 (13.49–17.78)], tacrolimus [ROR (95% CI):12.7 (11.57–13.95)], etonogestrel and ethinylestradiol vaginal ring [ROR (95% CI):11.88 (10.95–12.89)]. After categorizing the drugs, the strongest risk signal is sex hormones and modulators of the genital system [ROR (95% CI):11.88 (10.95–12.89)], followed by analgesics [ROR (95%CI): 6.73 (6.38–7.1)], immunosuppressants [ROR (95% CI):3.91 (3.76–4.05)], antineoplastic agents [ROR (95% CI):3.33 (3.22–3.45)], corticosteroids for systemic use [ROR (95% CI): 2.94 (2.73–3.18)], antiepileptics [ROR (95% CI):1.93 (1.71–2.18)], drugs used in diabetes [ROR (95% CI):1.5 (1.34–1.67)], antibacterials for systemic use [ROR (95% CI):1.46 (1.28–1.68)].

**Conclusion:**

Our findings indicate that multiple drugs are associated with an increased risk of coagulopathies. From the pharmacovigilance perspective, proactive analysis of these drugs aids in clinical monitoring and enhances risk identification of coagulopathies.

## 1 Introduction

Coagulopathies are diseases characterized by reduced blood clotting capacity, resulting in a pathological tendency toward bleeding and thrombosis (Iba et al., 2019a; Iba and Levy, 2020). Coagulopathies arise from various conditions, such as severe trauma, sepsis, cancer, hematological malignancies, and pregnancy-related complications (Levi, 2014; Lockhart et al., 2016; Iba et al., 2019b; Kleinveld et al., 2022). In addition, drug-induced coagulopathies are also common in clinical practice (Novak et al., 2012; Cui et al., 2019; Kumar et al., 2019). Coagulopathies can stem from platelet dysfunction, impaired thrombin generation, hypofibrinogenemia, and hyperfibrinolysis (Gando et al., 2016; Bartoszko and Karkouti, 2021). Coagulopathies are severe complications in patients, which can lead to multiple organ dysfunction and are linked to poorer patient prognosis (Helms et al., 2023; Li et al., 2023). Studies have shown that up to 56% of trauma patients and over 40% of critically-ill patients develop coagulopathies (Stensballe et al., 2017; Petros, 2019). Severe coagulopathies are associated with a more than fourfold increase in adverse bleeding events, blood transfusion volume, and mortality, along with prolonged hospital and ICU stays. This heightened risk highlights the importance of early identification and prevention of coagulopathies (Zhao et al., 2021; Li et al., 2023).

Clinical manifestations of coagulopathies are diverse, primarily including bleeding tendency (such as skin ecchymosis, joint hematoma, and visceral hemorrhage), thrombosis (e.g., deep vein thrombosis and pulmonary embolism). They are usually accompanied by abnormal laboratory results, such as prothrombin time (PT), activated partial thromboplastin time (APTT), fibrinogen concentration, platelet count, function testing, and special coagulation factor testing (Moore et al., 2020; Giustozzi et al., 2021; Gómez-Mesa et al., 2021). Drugs can affect the coagulation system through multiple biomolecules and signal pathways. For example, some drugs inhibit the activity of catalases, while others cause bleeding by interfering with the interaction between platelets and blood vessel walls. These complex mechanisms of action complicate the diagnosis and treatment of drug-induced coagulopathies (Cassar et al., 2005; Xiao et al., 2013; Izzedine and Perazella, 2015; Bauer et al., 2022). Due to the complex pathogenesis and diagnostic challenges of coagulopathies, which often require multidisciplinary collaboration, research in this area is limited. Identifying drugs closely linked to coagulopathies is essential, as it enables medical institutions to develop precise monitoring and intervention strategies while providing critical safety information for clinicians and pharmacists to support safer drug use for patients.

Currently, information on adverse reactions related to coagulopathies is mainly recorded in drug labels. Although drug safety is evaluated in clinical trials, these clinical trials may not fully capture the real-world scenario due to sample limits, treatment duration, and co-morbid conditions (Javed and Kumar, 2024). Therefore, conducting real-world research offers a more comprehensive approach to understanding drug safety. Although identifying drugs related to coagulopathies is crucial, no comprehensive list of these drugs currently exists. While most existing research focuses on evaluating the coagulopathy risks of individual drugs, it is equally important to investigate coagulopathies associated with a broader range of medications (Chai and Babu, 2014; Cui et al., 2019; Peralta et al., 2019; Guo et al., 2022).

Compared to laboratory and clinical trial data, pharmacovigilance data reflects real-world drug use more accurately and is vital for post-market surveillance (Raschi et al., 2020b). The FAERS is the largest public drug alert database for spontaneous reports of adverse events, gathering data from medical personnel, consumers, manufacturers, *etc.* It plays an essential role in informing healthcare professionals and the public about the potential risks of drugs (Sakaeda et al., 2013; Raschi et al., 2019; Raschi et al., 2020a).

A signal is new or previously unknown information linking an adverse event to a drug (Javed and Kumar, 2024). Generating a signal requires more than one high-quality report (Subeesh et al., 2017; Sharma and Kumar, 2022). Data mining algorithms (DMAs) such as reporting odds ratio (ROR), proportional reporting ratio (PRR), and Bayesian confidence propagation neural network (BCPNN) are common analytical methods for detecting signals in pharmacovigilance databases (Kubota et al., 2004; Rothman et al., 2004). These methods identify patterns of associations or unexpected occurrences of events in large databases using statistical analysis.

The purpose of this study is to comprehensively investigate the risks of drug-induced coagulopathies through the FAERS and identify drugs with potential coagulopathy risks that are not listed in the package insert. This research aims to provide an overview of drugs that may induce coagulopathies from a pharmacovigilance perspective, offering valuable insights for clinical practice.

## 2 Materials and methods

### 2.1 Data sources and processing procedures

The FAERS database is the central system for post-marketing adverse drug reaction monitoring in the United States and is also one of the main approaches for current pharmacovigilance research (Zhang et al., 2024). In our study, ASCII data packages submitted from the first quarter of 2004 to the second quarter of 2024 were retrieved from the database. Each package contains demographic and administrative information (DEMO), drug information (DRUG), adverse events (REAC), patient outcomes (OUTC), report sources (RPSR), start and end dates for reported drugs (THER), indications for use (INDI) (Yang et al., 2022). Import all data analysis into R version 4.3.3 and Excel software for data cleaning and analysis. Delete duplicate data according to the FDA’s suggestion. If the case has the same case ID, it will retain the latest report with FDA_DT; if the case ID is the same as the FDA_DT, it will retain a large master ID. After the repeated data is eliminated, some primary ID repeated items are still found, so the auxiliary duplicate data is performed (Yu et al., 2021).

The symptoms of AEs are coded using the Medical Dictionary for Regulatory Activities (MedDRA). MedDRA is an internationally standardized and clinically validated terminology system (Kumar et al., 2019). In the FAERS database, encode each report using the preferred term (PT) from MedDRA terminology, which is categorized into High-Level Term (HLT), High-Level Group Term (HLGT), and System Organ Class (SOC) in MedDRA (Zhang et al., 2023; Li et al., 2024). According to the latest MedDRA 27.0 version, our study searched for “coagulopathies (MedDRA 10064477)” at the HLT level and identified 26 related PTs, mainly including coagulopathy, disseminated intravascular coagulation, thrombotic microangiopathy, hypercoagulation, antiphospholipid, *etc.* In line with MedDRA 27.0 standards, only “primary suspect (PS)” drugs coded by PT were included to focus on the highest level of suspicion for drug-related coagulopathies. The Anatomical Therapeutic Chemical (ATC) classification system was used to code the preliminary drug list, the final drug list for analysis was obtained after excluding ambiguous drug names and integrating drugs with the same ingredient (Fan et al., 2024; Li et al., 2024).

### 2.2 Data analysis

In this study, we employed ROR, PRR, and BCPNN to identify signals for potential increased risk of drug-related coagulopathies (Poluzzi et al., 2009; Sakaeda et al., 2013; Sharma and Kumar, 2022; Zou et al., 2023; Javed and Kumar, 2024). The ROR and PRR algorithms are frequentist (non-Bayesian) algorithms, which are simple to calculate and have high sensitivity. The advantage of ROR is that it corrects for bias due to the low number of reports of certain events compared to PRR, while the advantage of PRR is that it is less affected by the omission of adverse events (Evans et al., 2001; Rothman et al., 2004). BCPNN, a Bayesian algorithm, effectively integrates data from multiple sources and supports cross-validation (Kubota et al., 2004). It accounts for uncertainties in the disproportionate rate, especially with smaller adverse event samples, reduces false positives, and is used for pattern recognition in higher dimensions (Tang et al., 2022). This study combines multiple algorithms to leverage their respective strengths, expanding the detection range and cross-validating results to enhance sensitivity and specificity in signal detection (Noguchi et al., 2018; Zhou et al., 2023). Higher values of these parameters indicate stronger signal strength, representing the level of association between drugs and coagulopathies. The formulas and criteria for each algorithm are shown in Table 1 (Sharma et al., 2023; Liu et al., 2024). Positive signals are identified if any of the three methods’ criteria are met, indicating a possible association between the drugs and the event (Alenzi et al., 2024).

**TABLE 1:** Summary of algorithms used for signal detection.

Measure	Calculation formula	Criteria
ROR	ROR = ad/bc	a≥3; lower limit of 95% CI > 1
	95%CI = e^ln(ROR)±1.96(1/a+1/b+1/c+1/d)^0.5^	
PRR	PRR = a (c + d)/c (a + b)	a≥3; PRR≥2, χ^2^ ≥ 4
	χ^2^ = [(ad-bc)^^^2](a+b + c + d)/[(a+b) (c + d) (a+c) (b + d)]	
BCPNN	IC = log2a (a+b + c + d)/[(a+c) (a+b)]	IC025 > 0
	95%CI = E (IC)±2 [V(IC)] ^ 0.5	

PRR, proportional reporting ratio; ROR, reporting odds ratio; BCPNN, bayesian confidence propagation neural network; a, number of reports with coagulation dysfunction caused by the target drug; b, number of reports with other AEs, caused by the target drug; c, number of reports with coagulation dysfunction caused by other drugs; d,number of reports with other AEs, caused by other drugs; CI, confidence interval; IC, information component; IC025, the lower limit of the 95% CI, of the IC; E (IC), the IC, expectations; V (IC), the variance of IC.

## 3 Results

### 3.1 Descriptive analysis

#### 3.1.1 The basic process for retrieving adverse event reports of target drugs

**FIGURE 1:**
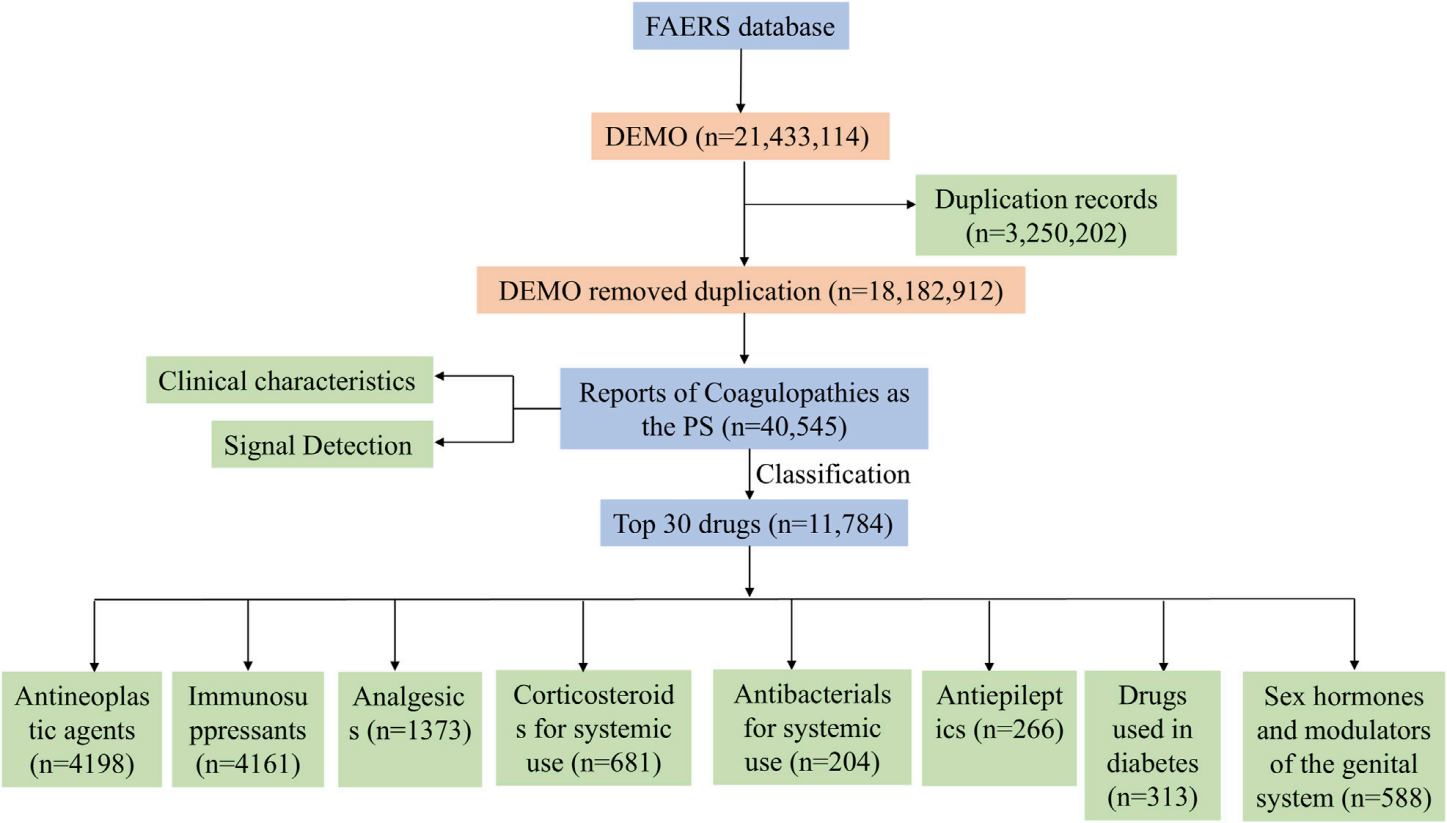
Flow chart for identifying suspected coagulopathies reports.

A total of 21, 433, 114 reports were retrieved from the FAERS database. After data cleaning and analysis, 40,545 reports on coagulopathies were collected. We found that 4,687 drugs are related to coagulopathies. After removing anticoagulant and antiplatelet drugs, we conducted a comprehensive analysis of the top 30 drugs, which is detailed in the flowchart (Figure 1).

#### 3.1.2 Clinical information on adverse event reports

**FIGURE 2:**
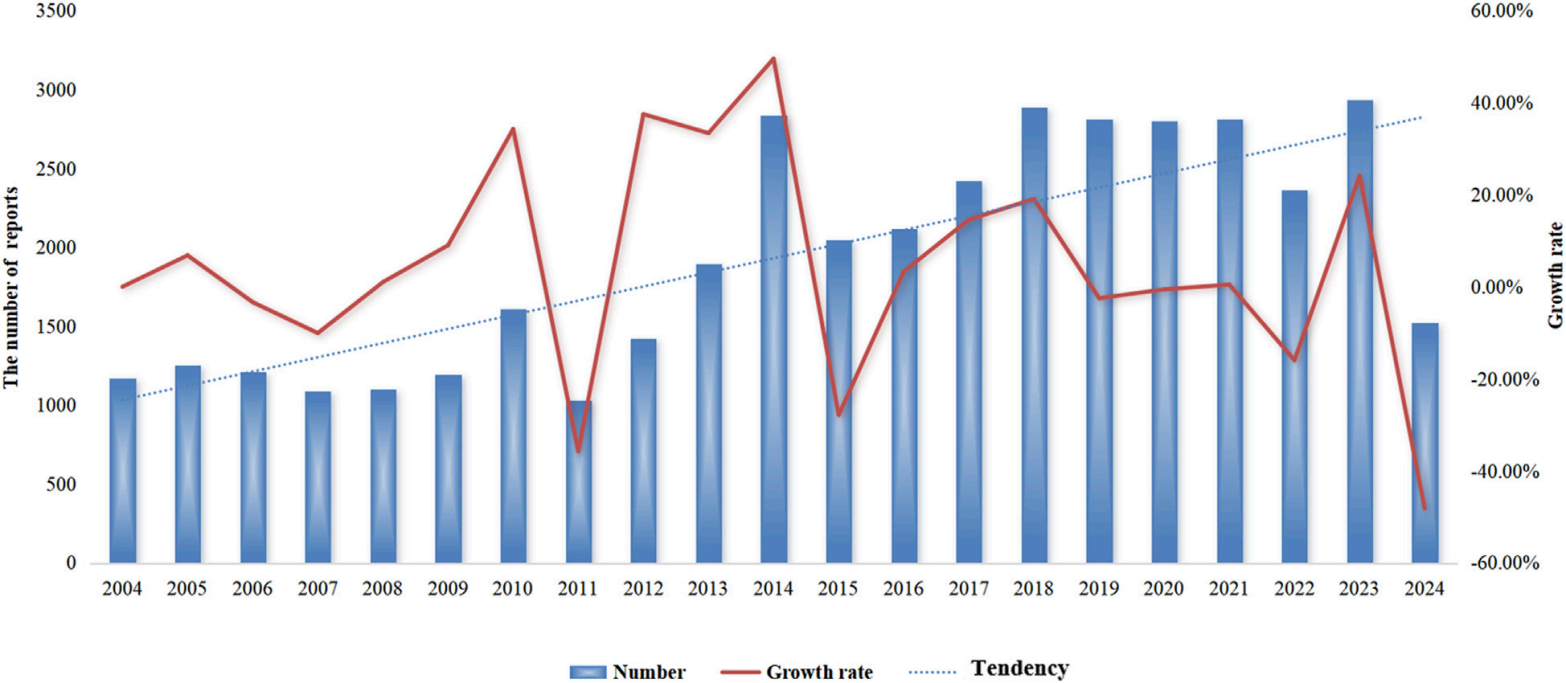
Annual trend in reporting of adverse drug events related to coagulopathies.

Detailed information on the adverse event reports of the patient was introduced in Table 2. As for patients, males reported 17,893 (44.1%) adverse event reports, while females submitted 18,734 (46.2%), with a slightly higher number of reports from females than males. Regarding age composition, patients aged 18–64.9 submitted 16,547 (40.8%) adverse event reports, accounting for the most significant proportion. These data come from submissions from many countries. The United States is the most extensive reporting country, and it submitted 14,951 (36.9%) reports, followed by Japan, which submitted 7,310 (18%) adverse event reports. In terms of the ending of the patient, the number of “deaths” in patients is the largest, with a total of 13,183 (32.5%) adverse event reports, followed by “hospitalization or prolongation of hospitalization” with 12,511 (30.9%) adverse event reports. In terms of reporting year, 2014 had the highest reported cases (Figure 2). After searching the PTs contained in “coagulopathies”, a total of 26 related PTs were obtained. Among them, “coagulopathy” is the most reported PT (Table 3).

**TABLE 2:** Clinical characteristics of reported drug-induced coagulopathies.

Characteristics	Reports, n (%)
Age	
<18	3,675 (9.1)
>85	974 (2.4)
18–64.9	16,547 (40.8)
65–85	10,419 (25.7)
Unknown	8,930 (22.0)
Gender	
Male	17,893 (44.1%)
Female	18,734 (46.2%)
Unknown	3,918 (9.7%)
Reporting country	
United States	14,951 (36.9)
Japan	7,310 (18)
France	1876 (4.6)
Germany	1,225 (3.0)
Spain	1,151 (2.8)
Others or unknown	14,032 (34.6)
Outcome	
Death	13,183 (32.5)
Hospitalization or prolongation of hospitalization	12,511 (30.9)
Life-threatening	4,558 (11.2)
Disability	180 (0.4)
Congenital anomaly	28 (0.1)
Others or unknown	10,085 (24.9)

**TABLE 3:** Number of drugs associated with PT group.

Group	Code	No. (%) of drugs
Coagulopathy	10,009802	15057 (35.84)
Disseminated Intravascular Coagulation	10,013442	12954 (30.83)
Thrombotic Microangiopathy	10079988	8,050 (19.16)
Hypercoagulation	10020608	1771 (4.22)
Antiphospholipid Syndrome	10002817	1,395 (3.32)
Factor Viii Inhibition	10048619	1,007 (2.40)
Hypocoagulable State	10020973	861 (2.05)
Abnormal Clotting Factor	10049862	198 (0.47)
Factor V Inhibition	10056335	180 (0.43)
Factor Ix Inhibition	1,0051778	127 (0.30)
Heparin Resistance	10059598	93 (0.22)
Hyperfibrinogenaemia	10051124	61 (0.15)
Coagulation Disorder Neonatal	10009732	55 (0.13)
Hyperfibrinolysis	10074737	52 (0.12)
Activated Protein C Resistance	10067648	33 (0.08)
Von Willebrand'S Factor Inhibition	10070690	30 (0.07)
Factor Xiii Inhibition	10059608	27 (0.06)
Disseminated Intravascular Coagulation In Newborn	10013443	25 (0.06)
Factor Vii Inhibition	10075240	16 (0.04)
Acquired Dysfibrinogenaemia	10051122	7 (0.02)
Lupus Anticoagulant Hypoprothrombinaemia Syndrome	10085219	4 (0.01)
Hyperprothrombinaemia	10067920	3 (0.01)
Hyperthrombinaemia	10058516	3 (0.01)
Factor Ii Inhibition	10075242	2 (0.00)
Factor X Inhibition	10075241	1 (0.00)
Pseudo-Heparin Resistance	10088924	1 (0.00)

### 3.2 Drugs that increase the risk of coagulopathies

**FIGURE 3:**
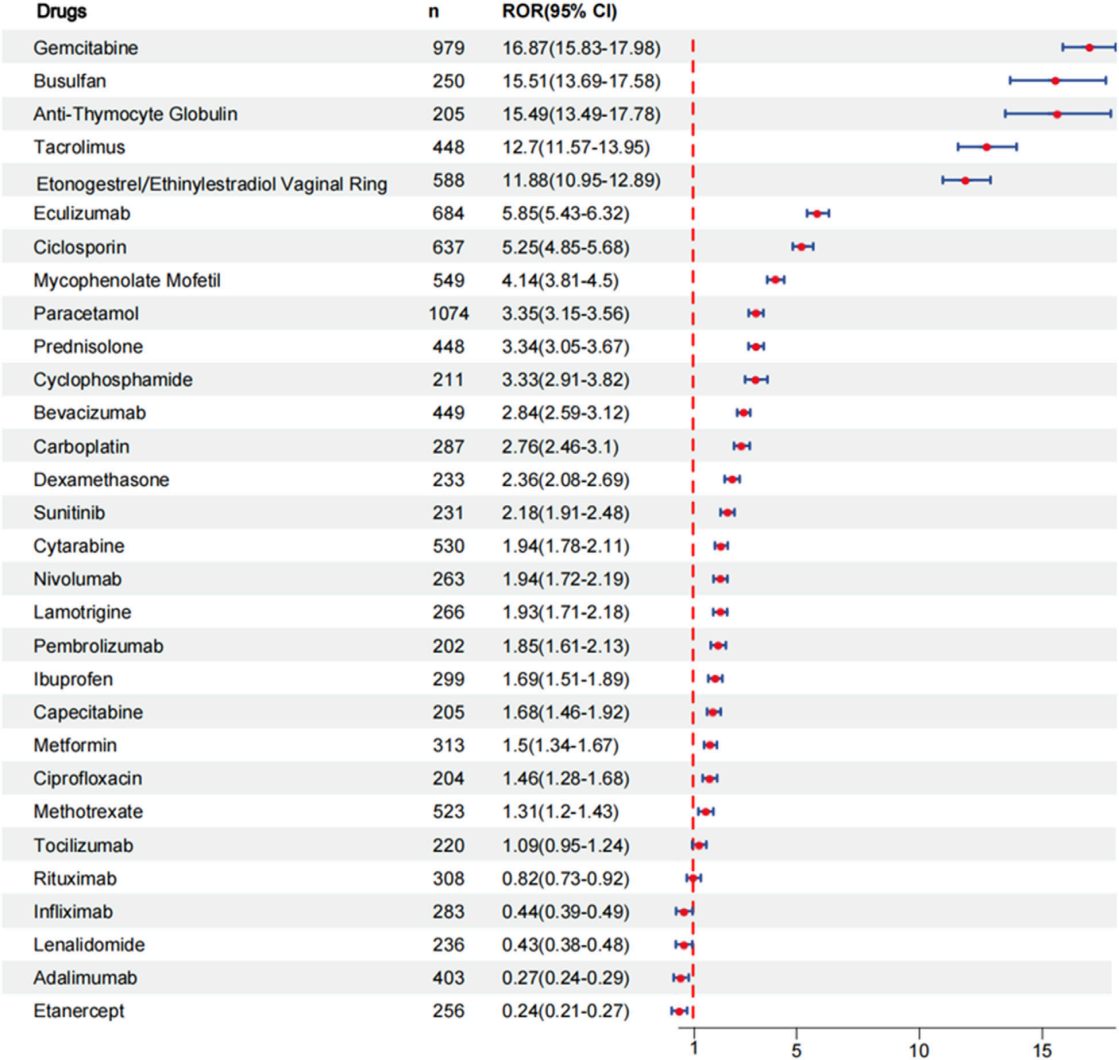
ROR for coagulopathies of single drug.

**FIGURE 4:**
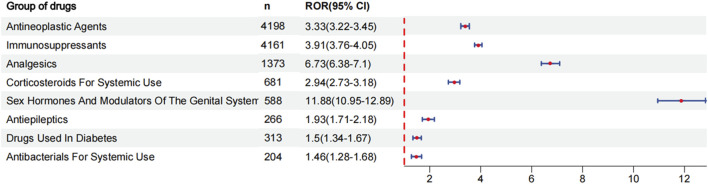
ROR for coagulopathies of each group of drugs.

To evaluate the risk signals associated with various drugs that cause coagulopathies, analyze the top 30 drugs with representative reported quantities. These drugs can be divided into the following categories: antineoplastic agents, immunosuppressants, analgesics, corticosteroids for systemic use, sex hormones and modulators of the genital system, antiepileptics, drugs used in diabetes, antibacterials for systemic use. We analyzed the risk signal intensity of individual drugs, and the specific analysis results are shown in Figure 3 and Supplementary Table S1. At the same time, we also evaluated the strength of risk signals after classification, and a comprehensive summary of the detailed analysis is provided in Figure 4 and Supplementary Table S2. To provide a more comprehensive profile of these top 30 drugs, we compiled a list of drugs containing indications, dose, mode of administration, and adverse effects based on drug labels, which are summarized in Supplementary Table S3.

#### 3.2.1 Single drug risk signal detection

From Supplementary Table S1, it can be seen that 24 drugs exhibit positive signals. The top 5 drugs with positive signals are gemcitabine [ROR (95% CI):16.87 (15.83–17.98)], busulfan [ROR (95% CI):15.51 (13.69–17.58)], anti-thymocyte globulin [ROR (95% CI):15.49 (13.49–17.78)], tacrolimus [ROR (95% CI):12.7 (11.57–13.95)], etonogestrel and ethinylestradiol vaginal ring [ROR (95% CI):11.88 (10.95–12.89)]. Other positive signal drugs are arranged in order of risk signal strength: eculizumab, ciclosporin, mycophenolate mofetil, paracetamol, prednisolone, cyclophosphamide, bevacizumab, carboplatin, dexamethasone, sunitinib, cytarabine, nivolumab, lamotrigine, pembrolizumab, ibuprofen, capecitabine, metformin, ciprofloxacin, methotrexate. The larger the value of the ROR, the stronger the risk signal, indicating a greater risk of causing coagulopathies. The higher the ROR value, the greater the possibility of adverse events related to the use of specific drugs.

#### 3.2.2 Risk signals after classification of drugs

After the drug classification, the ranking is based on ROR: sex hormones and modulators of the genital system [ROR (95% CI):11.88 (10.95–12.89)], analgesics [ROR (95%CI): 6.73 (6.38–7.1)], immunosuppressants [ROR (95% CI):3.91 (3.76–4.05)], antineoplastic agents [ROR (95% CI):3.33 (3.22–3.45)], corticosteroids for systemic use [ROR (95% CI): 2.94 (2.73–3.18)], antiepileptics [ROR (95% CI):1.93 (1.71–2.18)], drugs used in diabetes [ROR (95% CI):1.5 (1.34–1.67)], antibacterials for systemic use [ROR (95% CI):1.46 (1.28–1.68)]. The strongest risk signal is sex hormones and modulators of the genital system, followed by analgesics, immunosuppressants, antineoplastic agents, corticosteroids for systemic use, antiepileptics, drugs used in diabetes, antibacterials for systemic use.

## 4 Discussion

This study provides a comprehensive and systematic investigation of adverse events related to drug-induced coagulopathies using the FAERS database. We identified drugs significantly associated with coagulopathies based on case numbers and signal strength. We observed that some drugs do not list coagulopathy-related adverse reactions in their labeling, highlighting the need to further explore drugs closely associated with coagulopathies.

To minimize bias and reduce the occurrence of false positives and false negatives, we used ROR, PRR, and BCPNN methods for analysis. A total of 24 drugs exhibited positive signals in the risk analysis. In addition, standardized naming was used to ensure precise and reliable analysis of our findings. According to their pharmacological effects, the drugs with positive signals can be divided into the following categories: anti-tumor drugs, immunosuppressants, anti-inflammatory and analgesic drugs, hypoglycemic drugs, antiepileptic drugs, steroid hormone drugs, antibacterial drugs, and contraceptive drugs. Higher ROR values indicate a greater risk of coagulopathy-related adverse reactions. Our research provides a basis for clinicians to make informed prescribing decisions and serves as a reminder for healthcare professionals to be vigilant about potential coagulopathies of these drugs in clinical practice.

Anti-tumor drugs accounted for the largest proportion of coagulopathy cases in our study. The incidence of coagulopathies in cancer patients is approximately 6%–15%. With cancer increasingly managed as a chronic disease and the use of new drugs on the rise, their incidence rate is expected to rise (Lechner and Obermeier, 2012; Al-Nouri et al., 2015). Anti-tumor drug therapies appear to be more common contributors to coagulopathies than cancer itself, with associated conditions including thrombotic microangiopathy (TMA), thrombocytopenia, intravascular thrombosis, and ischemia-induced terminal organ damage (Izzedine and Perazella, 2015; Jodele et al., 2015). The chemotherapeutic drugs most frequently associated with coagulopathies are mitomycin-C and gemcitabine (Aklilu and Shirali, 2023). In our study, gemcitabine demonstrated the highest ROR value and risk intensity. Gemcitabine is a pyrimidine analog that promotes apoptosis in rapidly dividing cells by disrupting DNA synthesis (Pandit and Royzen, 2022). Numerous studies have reported gemcitabine-induced coagulopathies, and the use of gemcitabine may increase the incidence of cancer patients (Grall et al., 2021; Ishikawa et al., 2021; van der Heijden et al., 2023). While the mechanism of gemcitabine-induced coagulopathies remains unclear, it is hypothesized to involve microvascular endothelial damage and immune complex-mediated endothelial injury (Mini et al., 2006; Zupancic et al., 2007). Carboplatin, a second-generation platinum-based anticancer drug, is cell cycle non-specific and exerts anti-tumor effects by interfering with DNA synthesis and replication (Ettinger et al., 2022; Porter et al., 2023). It has been reported that carboplatin alone or in combination with other drugs can induce coagulopathies (Iams et al., 2013; Salhi et al., 2021). Given that many cancer patients are treated with multiple chemotherapy drugs, identifying the specific causative agent of coagulopathies can be challenging. Drug-induced coagulopathies may disrupt anti-tumor treatment in patients and increase the risk of cancer progression. Our study aims to discover drugs closely related to coagulopathies, which can help clinicians discover adverse drug reactions during treatment, support preventive measures and promote rational drug use in clinical practice.

Immunosuppressants-induced coagulopathies also accounted for a substantial proportion of cases in our study. Coagulopathies are a severe adverse reaction of immunosuppressants, mainly manifested as systemic platelet aggregation, thrombocytopenia, and mechanical damage to red blood cells (George and Nester, 2014). The mechanism behind immunosuppressant-induced coagulopathies may involve immune-mediated responses as well as dose- or duration-dependent toxicity (Bhavsar et al., 2016). Endothelial injury triggers the formation of microthrombi and platelet aggregation in the vascular system (Nwaba et al., 2013). Tacrolimus is a calcineurin inhibitor commonly used as an immunosuppressant post-solid organ transplants. There have been many reports on coagulopathies caused by tacrolimus (Nwaba et al., 2013; Cortina et al., 2015; Markan et al., 2021), and some have been fatal (Gill and Meghrajani, 2022). There are also some reports of coagulopathies caused by the combination of tacrolimus and ciclosporin; both drugs showed strong signals in our study (Pham et al., 2000). Recently, there have been reports that vascular endothelial growth factor (VEGF) inhibitors such as bevacizumab can lead to coagulopathies. VEGF stimulates signaling pathways and transcription by activating its receptor VEG-FR2, which is crucial for angiogenesis. Bevacizumab disrupts this pathway, leading to thrombotic microvascular disease (Apte et al., 2019; Estrada et al., 2019).

Our study reveals that sex hormones and reproductive system regulators have the highest risk of inducing coagulopathies. Compelling evidence suggests that female hormone replacement therapies and combination oral contraceptives can induce coagulopathies and increase the risk of venous thromboembolism (Watson, 2007). The formation of coagulopathies may be related to the increased plasma concentrations of procoagulant proteins, reduced anticoagulant proteins, and altered fibrinolysis (Gialeraki et al., 2017; Teal and Edelman, 2021). Studies have shown that patients with oral contraceptives may face a significantly increased risk of developing coagulopathies during the perioperative period (Watson, 2007). Especially for patients with compound oral contraceptives, the probability of occurrence of coagulopathies is 2–4 times higher compared to patients who do not use them (Haverinen et al., 2022). The risk of coagulopathies is dose-dependent on the dose of sex hormones used (Weill et al., 2016). Excessive use of antipyretic and analgesic drugs can also lead to coagulopathies, characterized by an increased prothrombin time and international normalized ratio (Larson et al., 2005; Habib et al., 2013). Some antibiotics can increase the risk of coagulopathies, which has been confirmed in multiple studies, such as cefoperazone sulbactam, tigecycline, and linezolid (Routsi et al., 2015; Hakeam et al., 2018; Cui et al., 2019; Treml et al., 2021; Wu et al., 2021). Our study found that ciprofloxacin is associated with coagulopathies, although few studies have been conducted.

In our study, we identified some (OTC) drugs, such as paracetamol and ibuprofen, as potentially associated with coagulopathies, even though they can be purchased without a prescription. Patients should carefully read instructions before using OTC drugs, strictly follow the recommended dosage and frequency, and avoid altering the dosage or administration method (Sánchez-Sánchez et al., 2021). If symptoms of coagulopathies appear, such as severe bleeding or uncontrollable bleeding, patients should discontinue use immediately and seek medical attention (Tan et al., 2020). Pharmacists should assess patients on their health status, allergy history, medication history, and any concurrent treatments that could influence coagulopathy risk when dispensing OTC drugs. Governments, medical institutions, and pharmaceutical manufacturers can collaborate to raise awareness about the adverse reactions associated with OTC drugs and help to enhance patients’ understanding of these potential risks (Gheorghe et al., 2019; Yousaf et al., 2020). Open communication between patients and healthcare providers is crucial to optimize patient care and ensure safety. Documenting the medical history of patient is a critical step in prevention, especially for those with a history of coagulopathies, who should exercise particular caution. For critically ill patients using these drugs, regular blood coagulation testing is particularly necessary (Song et al., 2020; Fan et al., 2024). In our study, monitoring cases of coagulopathies associated with any drug in the FAERS database was critical for helping clinicians properly diagnose and manage this condition. Comprehensive information enables healthcare providers to make informed treatment decisions and apply strategies to reduce the risk of drug-induced coagulopathies.

We found that many drugs with an elevated risk of coagulopathies, such as Mycophenolate Mofetil, Carboplatin, Cytarabine, Nivolumab, Metformin, Rituximab, and Infliximab, were not listed with coagulopathy risks in their instructions. Some drugs, such as Paracetamol, Prednisolone, Dexamethasone, and IbuProfen. In the instructions, only their use will affect the efficacy of oral anticoagulant drugs, but coagulopathies are not mentioned in adverse reactions. Although many drugs have been on the market for a long time, awareness of coagulopathy risks is limited, and data on the prevention and management of drug-induced coagulopathies remain sparse. Monitoring drug-induced coagulopathies is crucial, particularly for cancer and critically ill patients, as it may reduce mortality rates. With the advent of new drugs, especially anti-tumor drugs and immunosuppressants, continuous monitoring of drug-induced coagulopathies will better inform clinicians in making optimal treatment decisions.

This study has several limitations. First, the study established only the association between drugs and adverse events, while lacking definitive proof of the causal relationship between drug exposure and the reported event (Tobaiqy et al., 2010; Jain et al., 2023). Second, the FAERS data was based on spontaneous and voluntary reports, which may be influenced by recent research findings or media coverage (Fan et al., 2024; Jiang et al., 2024). Third, our study did not account for the impact of concomitant drugs and secondary suspect drugs (SS) on adverse reactions. Finally, this study did not assess whether coagulopathy-related adverse reactions are dose-dependent.

## 5 Conclusion

In summary, coagulopathies represent severe adverse reactions, with some cases leading to fatal outcomes, particularly among patients with severe illness and cancer. Despite the serious nature of coagulopathies, the risks have not been sufficiently assessed. In this study, we conducted a comprehensive evaluation of drugs related to coagulopathies using the FAERS database and systematically analyzed the top 30 drugs with strong risk signals. For clinical use, we recommend enhanced drug safety monitoring to reduce the risk of coagulopathies when administering these medications.

## Data Availability

The original contributions presented in the study are included in the article/Supplementary Material, further inquiries can be directed to the corresponding author.
