# Supplementary material for: Drug-induced coagulopathies: a real-world pharmacovigilance study using the FDA adverse event reporting system
Source: Front Pharmacol. 2024 Dec 18;15:1486422. doi: 10.3389/fphar.2024.1486422 (PMC11688381; doi:10.3389/fphar.2024.1486422)
Supplement: Supplementary file 2 [file Table3.pdf]

TABLES3. Drug list of the top 30 drugs

Drugs	Indications	Dose	Mode of administration	Adverse effects
Gemcitabine	Non-Small Cell Lung Cancer, Breast Cancer, Pancreatic Cancer, Ovarian Cancer.	Injection: 0.2g, 1g	Intravenous injection	Hematologictoxicity, Myelosuppression, Pulmonary Toxicityand Respiratory Failure, Hepatic Toxicity, Exacerbation of Radiation Therapy Toxicity, Capillary Leak Syndrome, Embryo- Fetal Toxicity, Posterior Reversible Encephalopathy Syndrome, Hemorrhage, Neutropenia, Neuropathy-Sensory, Anemia, Thrombocytopenia, Alopecia, Nausea, Vomiting, Diarrhea, etc.
Busulfan	Chronic myelogenous leukemia.	Injection: 60mg/10ml	Intravenous injection	Prolonged prothrombin time, Myelosuppression, Seizures, Hepatic Veno-Occlusive Disease, Embryo-fetal Toxicity, Cardiac Tamponade, Bronchopulmonary Dysplasia, Cellular Dysplasia, Fever, Headache, Asthenia, etc.
Anti-Thymocyte Globulin	Prophylaxis of acute rejection, Treatment of acute rejection.	Injection: 25mg/10ml	Intravenous injection	Low counts of platelets and white blood cells, Urinary tract infection, abdominal pain, Hypertension, Nausea, Shortness of breath, Fever, Headache, Aanxiety, Chills, Increased potassium levels in the blood.
Tacrolimus	Prophylaxis of Organ Rejection in Kidney, Heart Transplant, Liver.	Cream: 0.03%,0.1%<br>Capsule: 500ug,1mg, 5mg<br>Injection: 1ml/5mg	Oral<br>Intravenous injection<br>Cutaneous	Thrombotic Microangiopathy, Including Hemolytic Uremic Syndrome and Thrombotic Thrombocytopenic Purpura, Lymphoma and Other Malignancies, Serious Infections, Nephrotoxicity, Neurotoxicity, Hyperkalemia, Hypertension, etc.
Etonogestrel and Ethinylestradiol Vaginal Ring	Prevent pregnancy.	11.7mg etonogestrel<br>2.7mg ethinyl estradiol	Inserted in the vagina	Thromboembolic Disorders and Other Vascular Problems, Toxic Shock Syndrome (TSS), Liver Disease,High Blood Pressure, Hypersensitivity Reactions, Gallbladder Disease, etc.
Eculizumab	Paroxysmal Nocturnal Hemoglobinuria, Myasthenia Gravis, Neuromyelitis Optica Spectrum Disorder, Atypical Hemolytic Uremic Syndrome, Generalized.	Injection: 300mg/ 30mL	Intravenous injection	Thrombosis, Serious Meningococcal Infections, Headache, Nasopharyngitis, Back pain, Nausea, Fatigue, Cough, Herpes simplex infections, Sinusitis, etc.
Ciclosporin	Organ rejection in kidney, liver, and heart allogeneic transplants.	Capsule: 25mg, 100mg<br>Solution: 50ml/5g<br>Injection:	Oral<br>Intravenous injection	Thrombotic Microangiopathy, Nephrotoxicity, Hyperkalemia, Hepatotoxicity, Malignancies, Serious Infections, Polyoma Virus Infections, Neurotoxicity, etc.

		5ml,250mg		
Mycophenolate Mofetil	The prophylaxis of organ rejection.	Capsule: 250mg, Injection: 500mg	Oral Intravenous injection	Embryofetal Toxicity, Lymphoma and Other Malignancies, Serious Infections, Blood Dyscrasias: Neutropenia and Pure Red Cell Aplasia (PRCA), Inflammatory Syndrome (fever, arthralgias, arthritis, muscle pain and elevated inflammatory markers), Gastrointestinal Complications, etc.
Paracetamol	The management of mild to moderate pain in adult and pediatric patients 2 years and older, severe pain, fever.	Tablets: 0.3g, 0.5g, Capsule: 250mg, 100mg, Injection: 2ml, 0.25g.	Oral, Intramuscular injection,	Hepatic Injury, Serious Skin Reactions, Nausea, Vomiting, Hypersensitivity and anaphylaxis.
Prednisolone	Allergic States, Dermatologic Diseases, Endocrine Disorders, Gastrointestinal Diseases Hematologic Disorders, Miscellaneous, Nervous System, Ophthalmic Diseases, Renal Diseases, Respiratory Diseases, Rheumatic Disorders.	Tablets: 2.5mg,5mg, 10mg,20mg and 50 mg Injection: 40mg/ml, 125mg/2ml	Oral Intramuscular injection	Fluid and Electrolyte Disturbances, Musculoskeletal, Endocrine, Gastrointestinal, Dermatologic, Metabolic, Neurological, Ophthalmic, Urticaria and other allergic, Anaphylactic or hypersensitivity reactions.
Cyclophosphamide	Malignant Diseases, malignant lymphomas, Hodgkin's disease, mixed-cell type lymphoma, histiocytic lymphoma, Burkitt's lymphoma, multiple myeloma, leukemias, mycosis fungoides, neuroblastoma.	Tablets, Capsule:25 mg, 50mg Injection: 200mg/ml	Oral Intravenous injection	Thrombotic microangiopathy, Myelosuppression, Urinary Tract and Renal Toxicity, Immunosuppression, Bone Marrow Failure and Infections, Cardiotoxicity, Pulmonary Toxicity, Veno-occlusive Liver Disease, Secondary Malignancies, Embryo-Fetal Toxicity, Infertility, Impairment of Wound Healing, Hyponatremia.
Bevacizumab	Metastatic Colorectal Cancer, First-Line Non Squamous Non Small Cell Lung Cancer, Recurrent Glioblastoma, Metastatic Renal Cell	Injection: 100mg/4mL 400mg/ 16mL	Intravenous injection	Hemorrhage, Arterial Thromboembolic Events, Gastrointestinal Perforations and Fistulae, Venous Thromboembolic Events, Posterior Reversible Encephalopathy Syndrome, Hypertension, Surgery and Wound Healing Complications, Renal Injury and Proteinuria, Infusion-Related Reactions, Embryo-Fetal Toxicity, Ovarian Failure, Congestive Heart Failure (CHF).

	Carcinoma,<br>Persistent, Recurrent.			
Carboplatin	Cyclophosphamide for Initial Treatment of Ovarian Cancer,<br>Single Agent for Secondary Treatment of Advanced Ovarian Cancer.	Injection:<br>50mg/5mL<br>50mg/15mL<br>450mg/45mL,<br>600mg/60mL	Intravenous injection	Thrombocytopenia, Neutropenia, Leukopenia, Anemia, Infections, Bleeding, Transfusions, Nausea and vomiting, SGOT elevations, Vomiting, Neurologic, Serum creatinine elevations, Blood urea elevations, Bilirubin elevations, Sodium, Potassium, Pain, Asthenia, Cardiovascular, etc.
Dexamethasone	Allergic States,<br>Dermatologic Diseases,<br>Endocrine Disorders,<br>Hematologic Disorders,<br>Miscellaneous,<br>Neoplastic Diseases,<br>Nervous System,<br>Ophthalmic Disease,<br>Renal Diseases,<br>Respiratory Diseases,<br>Rheumatic Disorders.	Tablets:<br>0.5mg, 0.75 mg, 1.5 mg, 4 mg, 6mg<br>Injection:<br>2mg/ml	Oral<br>Intramuscular injection	Immunosuppression and Increased Risk of Infection, Varicella Zoster and Measles Viral Infections, Fungal Infections, Hepatitis B Virus Reactivation, Amebiasis, Strongyloides Infestation, Cardio-renal, Cerebral Malaria, Infections, Ophthalmic, Kaposi's Sarcoma
Sunitinib	Gastrointestinal stromal tumor (GIST),<br>Renal cell carcinoma,<br>Pancreatic neuroendocrine tumors (pNET).	Capsule:<br>12.5mg,<br>25mg,<br>37.5mg,<br>50mg	Oral	Thrombotic microangiopathy (TMA), Hepatotoxicity, QT Interval Prolongation and Torsade de Pointes, Cardiovascular Events, Hypertension, Hemorrhagic Events, Tumor Lysis Syndrome, Proteinuria, Dermatologic Toxicities, Thyroid Dysfunction, Impaired Wound Healing, Embryo-Fetal Toxicity.
Cytarabine	acute non-lymphocytic leukemia,<br>acute lymphocytic leukemia and the blast phase of chronic myelocytic leukemia,<br>meningeal leukemia.	Injection:<br>20mg/mL	Intravenous injection,<br>Intramuscular injection	Bone marrow suppressant, Anemia, Leukopenia, Reduced reticulocytes, Nausea, Vomiting, Diarrhea and abdominal pain, Oral ulceration, Thrombocytopenia, Megaloblastosis, Hepatic dysfunction.
Nivolumab	Melanoma,<br>Non-Small Cell Lung Cancer (NSCLC),<br>Malignant Pleural Mesothelioma,<br>Renal Cell Carcinoma,<br>Classical Hodgkin Lymphoma (cHL),<br>Squamous Cell	Injection:<br>40mg/4mL,<br>100mg/10mL,<br>120mg/12mL,<br>240mg/24 mL	Intravenous injection	Immune-Mediated Adverse Reactions, Fatigue, Rash, Pruritus, Musculoskeletal pain, Diarrhea, Nnausea, Asthenia, Cough, Dyspnea, Constipation, Decreased appetite, Back pain, Arthralgia, Headache, Upper respiratory tract infection, Pyrexia, Urinary tract infection, Abdominal pain, Vomiting, Hypothyroidism.

	Urothelial Carcinoma,<br>Colorectal Cancer.			
Lamotrigine	Epilepsy-adjunctive therapy,<br>Epilepsy-monotherapy,<br>Bipolar disorder.	Tablets:<br>25mg,<br>50mg,<br>100mg,<br>150mg,<br>200 mg,<br>250 mg	Oral	Fibrin decrease, Fibrinogen decrease, Thrombocytopenia., Allergic reaction, Chills, Malaise, Flushing, Hot flashes, Hypertension, Acne, Alopecia, Hirsutism, Eructation, Gastritis, Confusion, Paresthesia, Amblyopia, etc.
Pembrolizumab	Melanoma,<br>Non-Small Cell Lung Cancer,<br>Malignant Pleural Mesothelioma,<br>Classical Hodgkin Lymphoma,<br>Urothelial Cancer,<br>Primary Mediastinal<br>Gastric Cancer,<br>Esophageal Cancer,<br>Cervical Cancer, etc.	Injection:<br>100mg/4mL	Intravenous injection	Increased prothrombin INR, Immune-Mediated Adverse Reactions, Infusion-related reactions, Complications of allogeneic HSCT, Embryo-Fetal toxicity, fatigue, Musculoskeletal pain, Rash, diarrhea, Pyrexia, Cough, Decreased appetite, Pruritus, Dyspnea, Constipation, Pain, Abdominal pain, Nausea, Hypothyroidism, etc.
Ibuprofen	reduction of fever,<br>relief of mild to moderate pain,<br>relief of signs and symptoms of juvenile arthritis.	Tablets:<br>200mg<br>Suspension:<br>100mg/5ml	Oral	Cardiovascular Thrombotic Events, Gastrointestinal Risk, Serious Skin Reactions, including DRESS, Heart Failure and Edema, GI discomforthepatotoxicity, Anaphylactoid reaction, Fetal Toxicity.
Capecitabine	Colorectal Cancer,<br>Breast Cancer.	Tablets:<br>150mg<br>500 mg	Oral	Coagulopathy, Diarrhea, Cardiotoxicity, Dehydrationand Renal Failure, Embryo-Fetal Toxicity, Mucocutaneousand Dermatologic Toxicity, Hyperbilirubinemia, Hematologic.
Metformin	adjunct to diet and exercise to improve glycemic control in adults with type 2 diabetes mellitus.	Tablets:<br>500mg,<br>750mg<br>Extended Release Tablets:<br>500mg<br>1,000 mg	Oral	Lactic Acidosis, Vitamin B12 Deficiency, Hypoglycemia with Concomitant Use with Insulin and Insulin Secretagogues, Macrovascular Outcomes, Diarrhea, Nausea/Vomiting.
Ciprofloxacin	Skin and Skin Structure Infections,<br>Bone and Joint Infections,<br>Complicated Intra-Abdominal	Tablets:<br>250mg<br>500mg<br>750mg<br>Injection:<br>200mg/	Oral<br>Intravenous injection	Prothrombin time prolongation or decrease Cholesterol elevation, Tendinopathy and Tendon Rupture, Exacerbation of Myasthenia Gravis, Hypersensitivity Reactions, Other Serious and Sometimes Fatal Reactions, Hepatotoxicity, Serious Adverse Reactions with Concomitant Theophylline, Central Nervous System Effects, Clostridium Difficile-Associated Diarrhea, etc.

	Infections, Nosocomial, etc.	100mL		
Methotrexate	Neoplastic Diseases, Rheumatoid Arthritis, Polyarticular Juvenile Idiopathic Arthritis, Psoriasis.	Tablets: 2.5mg, Injection: 50mg/2mL, 1g/40mL.	Oral Intravenous injection Intramuscular injection	Thromboembolic events, Arterial thrombosis, Cerebral thrombosis, Deep vein thrombosis, Retinal vein thrombosis, Thrombophlebitis, Hypersensitivity Reactions, Embryo-Fetal Toxicity, Folic Acid Supplementation, Myelosuppression, Gastrointestinal Toxicity, Hepatotoxicity, Pulmonary Toxicity, Dermatologic Reactions, Renal Toxicity, Serious Infections, Neurotoxicity, Tumor Lysis Syndrome etc.
Tocilizumab	Rheumatoid Arthritis, Giant Cell Arteritis, Systemic Sclerosis-Associated Interstitial Lung Disease, Polyarticular Juvenile Idiopathic Arthritis, Systemic Juvenile Idiopathic Arthritis, Cytokine Release Syndrome, Coronavirus Disease 2019.	Injection: 80mg/4mL, 200mg/ 10mL, 400mg/ 20mL, 162mg/ 0.9mL	Intravenous injection Subcutaneous injection	Serious Infections, Gastrointestinal Perforations, Hepatotoxicity, Changes in Laboratory Parameters, Demyelinating Disorders, Immunosuppression, Hypersensitivity Reactions, Including Anaphylaxis, etc.
Rituximab	Non–Hodgkin's Lymphoma (NHL), Chronic Lymphocytic Leukemia (CLL), Rheumatoid Arthritis, Granulomatosis with Polyangiitis (GPA), Microscopic Polyangiitis (MPA), Pemphigus Vulgaris.	Injection: 100mg/ 10mL, 500mg/ 50mL	Intravenous infusion	Severe Mucocutaneous Reactions, Hepatitis B Virus Reactivation, B-Cell Malignancies, Infusion-Related Reactions, Cytopenias and hypogammaglobulinemia, Infections, Relapsed or Refractory, Low-Grade NHL, etc.
Infliximab	Crohn's Disease, Pediatric Crohn's Disease, Ulcerative Colitis, Pediatric Ulcerative Colitis, Psoriatic Arthritis, Plaque Psoriasis.	Injection: 100 mg	Intravenous infusion	Serious Infections, Malignancies, Hepatitis B Virus Reactivation, Hepatotoxicity, Heart Failure, Autoimmunity, Hematologic Reactions, Hypersensitivity, Neurologic Reactions, Concurrent Administration with Other Biological Products etc.
Lenalidomide	Multiple Myeloma, Myelodysplastic Syndromes, Mantle Cell Lymphoma,	Capsule: 2.5mg, 5mg, 10mg,15mg, 20mg, 25mg	Oral	Venous and Arterial Thromboembolism, Embryo-Fetal Toxicity, Lenalidomide REMS Program, Hematologic Toxicity, Increased Mortality in Patients with CLL, Second Primary Malignancies, Hepatotoxicity, Tumor Flare Reaction, Severe Cutaneous

	Follicular Lymphoma.			Reactions, Tumor Lysis Syndrome, Thyroid Disorders, Hypersensitivity.
Adalimumab	Rheumatoid Arthritis, Juvenile Idiopathic Arthritis, Crohn's Disease, Ulcerative Colitis, Plaque Psoriasis, Uveitis.	Injection: 40mg/ 0.8mL	Subcutaneous injection	Thrombosis, Serious Infections, Malignancies, Hypersensitivity Reactions, Hepatitis B Virus Reactivation, Neurologic Reactions, Hematological Reactions, Increased Risk of Infection when Used with Anakinra, Heart Failure, Autoimmunity, Immunizations, Increased Risk of Infection When Used with Abatacept.
Etanercept	Rheumatoid Arthritis, Polyarticular Juvenile Idiopathic Arthritis, Psoriatic Arthritis, Ankylosing Spondylitis, Plaque Psoriasis, Juvenile Psoriatic Arthritis.	Injection: 25mg/ 0.5mL, 50mg/mL	Subcutaneous injection	Serious Infections, Neurologic Reactions, Malignancies, New Onset or Worsening of Heart Failure, Hematologic Reactions, Hepatitis B Reactivation, Allergic Reactions, Immunizations, Autoimmunity, Immunosuppression.
